# Predictive Role of hsCRP in Recurrent Stroke Differed According to Severity of Cerebrovascular Disease: Analysis from a Prospective Cohort Study

**DOI:** 10.3390/jcm12041676

**Published:** 2023-02-20

**Authors:** Jiejie Li, Yuesong Pan, Mengxing Wang, Xia Meng, Jinxi Lin, Zixiao Li, Hao Li, Yilong Wang, Xingquan Zhao, Liping Liu, Yongjun Wang

**Affiliations:** 1 Department of Neurology, Beijing Tiantan Hospital, Capital Medical University, Beijing 100070, China; 2 China National Clinical Research Center for Neurological Diseases, Beijing 100070, China; 3 Advanced Innovation Center for Human Brain Protection, Capital Medical University, Beijing 100070, China; 4 Clinical Center for Precision Medicine in Stroke, Capital Medical University, Beijing 100070, China

**Keywords:** stroke, transient ischemic attack, prognosis, biomarkers, inflammation

## Abstract

Elevated levels of high-sensitivity C-reactive protein (hsCRP) were associated with an increased risk of recurrent stroke. However, it is still unknown whether the predictive value of hsCRP differed according to the severity of cerebrovascular disease. We used the cohort of the prospective multicenter cohort study of the Third China National Stroke Registry (CNSR-III), in which 10,765 consecutive patients with acute ischemic stroke or transient ischemic attack (TIA) had hsCRP levels measured. Patients were classified into minor stroke, or TIA, and non-minor stroke. The primary outcome was a new stroke within 1 year. Cox proportional hazards models were used to assess the association of hsCRP and its outcome. Elevated levels of hsCRP were associated with an increased risk of recurrent stroke in minor stroke or TIA patients, irrespective of using a National Institutes of Health Stroke Scale (NIHSS) score of ≤3 (the highest quartile vs. the lowest quartile: adjusted hazard ratio, 1.48; 95% CI, 1.12–1.97; *p* = 0.007) or ≤5 (the highest quartile vs. the lowest quartile: adjusted hazard ratio, 1.45; 95% CI, 1.15–1.84; *p* = 0.002) to define minor stroke. Such association was more apparent in the large-artery atherosclerosis subtype. However, for the patients with non-minor stroke, the association of hsCRP with recurrent stroke disappeared.

## 1. Introduction

Ischemic stroke and transient ischemic attack (TIA) are characterized by early recurrence, which may be fatal or disabling. The rate of recurrent stroke is reported to be around 10% within 3 months after the index event [1,2,3]. Early identification of patients at high-risk of recurrence is of clinical importance.

Inflammation contributes to the residual risk of recurrent stroke [4,5]. High-sensitive C-reactive protein (hsCRP), a well-known inflammatory marker, was shown to be potential in predicting the risk of recurrent stroke among patients with ischemic stroke or TIA in our and others’ previous studies [6,7,8,9,10,11,12,13,14,15,16,17]. High similarities between TIA and minor stroke in patients’ clinical characteristics and the more unstable status for recurrence suggested to dispense with distinction between them, and some large clinical trials have been initiated for early intensive secondary prevention in these high-risk patients [4,18,19,20,21]. Furthermore, we previously showed that hsCRP helped to stratify the risk of recurrent stroke in patients with TIA and minor stroke [22]. However, as an acute-phase protein, CRP was correlated with brain damage, which was directly reflected by severity of stroke or TIA clinically [23]. Although a few studies assessed the association between hsCRP and stroke recurrence, it remains unclear whether the predictive effect of hsCRP on recurrent stroke differed according to the severity of stroke. 

Using data from the Third China National Stroke Registry (CNSR-III), we investigate whether the association between hsCRP and recurrent stroke is affected by the severity of cerebrovascular disease. 

## 2. Methods

### 2.1. Study Design and Participants

The design of the CNSR-III has been described previously in detail [24]. Briefly, the CNSR-III was a nationwide prospective registry performed within seven days that included patients with acute ischemic stroke and TIA. Among 201 study sites from 22 provinces and 4 municipalities, 171 (85%) prespecified centers voluntarily participated in the pre-specified biomarker sub-study. The CNSR-III was approved by the ethics committee at Beijing Tiantan Hospital and participating centers. All participants or their legal proxies provided written informed consent.

### 2.2. Baseline Data Collection

At each study site, information on the participants’ age, sex, body mass index (BMI, the weight in kilograms divided by the square of the height in meters), cigarette-smoking history, medical history of hypertension, hypercholesterolemia, diabetes, ischemic stroke and coronary heart disease, and NIHSS score were collected at admission through a direct interview by trained research coordinators. We performed centralized etiology classification of ischemic stroke according to the TOAST (Trial of ORG 10,172 in Acute Stroke Treatment) criteria as described before [25,26].

### 2.3. Sample Collection and Measurement of hsCRP

Fasting blood specimens were collected within 24 h after enrollment. The median time of sampling was 55 h (interquartile range: 27–96 h) after index event onset. All specimens were extracted and transported through cold chain to the core laboratory in Beijing Tiantan Hospital. All specimens were stored at −80 ℃. Assays were performed centrally and blindly. Serum hsCRP was centrally measured on a Roche Cobas C701 analyzer with a lower limit of quantitation of 0.15 mg/L. 

### 2.4. Follow-Up and Outcomes

Participants were followed up by face-to-face or telephone interviews at the 1-year mark by trained research personnel, collecting information on stroke recurrence and cardiovascular events during the follow-up period. Confirmation of recurrent stroke was sought from the treating hospital, and suspected recurrence without hospitalization was judged by the independent endpoint judgment committee. The primary outcome was a new stroke event (ischemic or hemorrhagic) within 1 year. The secondary outcome was death within 1 year.

### 2.5. Statistical Analysis

The distribution of hsCRP was skewed. We presented categorical variables as percentages and continuous variables as medians with interquartile ranges. Skewed continuous variables and ordinal variables were compared with the Kruskal–Wallis test. Chi square statistic or Fisher exact test was used for comparisons of categorical variables as appropriate.

While minor stroke was of clinical importance, it was defined through various definitions. A National Institutes of Health Stroke Scale (NIHSS) score of ≤3 [4,19,20] or ≤5 [27,28,29] was the most frequently used one. Minor stroke was therefore defined by two criteria separately in the current study: a NIHSS score of ≤3 or ≤5 at the time of admission [22,30,31]. The hsCRP level was assessed according to quartiles: quartile 1: <0.82 mg/L, quartile 2: 0.82 to 1.78 mg/L, quartile 3:1.78 to 4.72 mg/L, and quartile 4: >4.72 mg/L, or as a continuous variable after logarithm transformation. In addition, the Justification for the Use of Statins in Prevention: an Intervention Trial Evaluating Rosuvastatin (JUPITER) study and the Canakinumab Anti-inflammatory Thrombosis Outcome Study (CANTOS) trial defined high-risk patients through the hsCRP cutoff level of ≥2 mg/L, which we therefore used as our cut-point as well [32,33]. The associations of hsCRP with recurrence and death were analyzed with the use of multivariable Cox proportional hazards models and logistic regression models. The potential confounders were age, sex, body mass index, smoking history, index event, medical histories of atrial fibrillation, coronary heart disease, ischemic stroke, diabetes, hypertension and hypercholesterolemia, baseline NIHSS score, baseline leukocyte count and low-density lipoprotein cholesterol level, usage of antiplatelet, antihypertensive, hypoglycemic and statin during a 1-year follow-up period, TOAST subtype, onset-to-sample collection time, intravenous recombinant tissue plasminogen activator treatment, and endovascular therapy. The unadjusted and adjusted hazard ratios (HRs) and their 95% confidence intervals (CIs) were calculated. A two-sided *p* value of <0.05 was considered to indicate statistical significance. SAS software, version 9.4 (SAS Institute, Inc., Cary, NC, USA) was used for all statistical analyses.

## 3. Results

### 3.1. Patient Characteristics

In the biomarker sub-study of CNSR-III, 10,765 (71.0%) patients with ischemic stroke or TIA had hsCRP measurement. Among them, 5055 and 7073 patients had minor stroke by using definitions of NIHSS score ≤3 and ≤5, and 735 patients had TIA. A total of 263 (2.4%) patients were lost to follow-up within one year. The baseline characteristics mostly differed between the patients with non-minor stroke and those with minor stroke (NIHSS ≤3 or ≤5) and TIA (Supplementary Table S1). The patients with minor stroke (NIHSS ≤ 3) or TIA and higher levels of hsCRP were significantly older and female, had higher BMI and baseline NIHSS score and LDL-C level, had histories of ischemic stroke, diabetes, hypertension, hypercholesterolemia, coronary heart disease and atrial fibrillation and minor stroke as a qualifying event, and received endovascular therapy and antihypertensive and hypoglycemic treatment during the follow-up (Table 1). Similar characteristics were observed when using the definition of NIHSS ≤ 5 to define minor stroke (Table 1).

### 3.2. Associations between hsCRP and Recurrent Stroke

A total of 485 (8.4%) and 715 (9.2%) patients, respectively, using NIHSS ≤ 3 and NIHSS ≤ 5 definitions, had subsequent stroke within 1 year (*p* = 0.12).

The elevated level of hsCRP was correlated with the incremental risk of recurrent stroke in the whole cohort (Table 2). When classifying patients according to severity, after adjustment for potential confounding factors, including onset-to-sample collection time and rt-PA and endovascular treatment, which was shown to affect hsCRP levels [34] and the predictive role of hsCRP in recurrent stroke or death [35,36], a consistent association between hsCRP and recurrence was observed in the patients with minor stroke or TIA, irrespective of using NIHSS score of ≤3 (the highest quartiles vs. the lowest quartiles: adjusted HR, 1.48; 95% CI, 1.11–1.96; *p* = 0.007; ≥2 mg/L vs. <2 mg/L: adjusted HR, 1.29; 95% CI, 1.06–1.57; *p* = 0.01; continuous model: adjusted HR, 1.13; 95% CI, 1.05–1.23; *p* = 0.001) or ≤5 (the highest quartiles vs. the lowest quartiles: adjusted HR, 1.45; 95% CI, 1.14–1.83; *p* = 0.002; ≥2 mg/L vs. <2 mg/L: adjusted HR, 1.24; 95% CI, 1.05–1.46; *p* = 0.01; continuous model: adjusted HR, 1.13; 95% CI, 1.06–1.20; *p* = 0.0002) to define minor stroke. However, for the patients with non-minor stroke, the association of hsCRP with recurrent stroke disappeared after adjustment. (Table 2)

The association of hsCRP with recurrent stroke was therefore further analyzed according to TOAST subtypes in the patients with minor stroke or TIA. An increased level of hsCRP was associated with recurrence in large-artery atherosclerosis subtype but not others, more apparently when using NIHSS score ≤5 definition (Table 3 and Supplementary Table S2).

### 3.3. Associations between hsCRP and Death

In total, 361 (3.4%) patients died within 1 year. Patients with higher hsCRP levels had an increased risk of death (Supplementary Table S3). Such association persisted in the patients with minor stroke or TIA, and non-minor stroke (Supplementary Table S3).

## 4. Discussion

In the current study, we found that the predictive role of hsCRP in recurrent stroke differed according to the severity of cerebrovascular disease. An elevated hsCRP level was correlated with an increased risk of recurrent stroke in the patients with TIA or minor stroke, irrespective of using NIHSS score of ≤3 or ≤5 to define minor stroke. However, such a relationship was attenuated in the patients with non-minor stroke. The association between hsCRP level and dependence or death appeared to maintain, regardless of severity.

Contrary to a line of research illustrating the association between hsCRP and recurrence after stroke, few studies assessed its role in recurrent stroke in specific high-risk patients with minor stroke or TIA, and yielded conflicting results [22,37]. Difference in the sample size and the definition of minor stroke might explain the controversy. For practical purposes, the NIHSS score was utilized as the evaluation scale to determine the severity of stroke. Various definitions of minor stroke has been proposed, among which NIHSS score ≤3 had the best outcomes [38]. Consistent with our prior study using NIHSS scores ≤3 [15], we found a significant relationship between hsCRP and recurrent stroke in the patients with TIA or minor stroke in the current study. Besides that, NIHSS score ≤5 was another commonly used definition of minor stroke [27,28,29]. In addition to proving our previous research, which showed that the two definitions had comparable clinical characteristics [39], our present study further added evidence that hsCRP had similar predictive values in stroke recurrence using these two definitions. However, such a relationship was apparently attenuated in the patients with non-minor stroke.

Atherosclerosis is a complex inflammatory disease [40]. CRP is an acute-phase protein produced in the liver, and also reflects chronic inflammation, including atherosclerosis burden [41]. Besides that, CRP has been shown to participate in inflammation response after acute stroke via modulating plaque instability, platelet activity, and thrombosis [42], which eventually leads to recurrent vascular events [43]. The relationship between CRP and recurrent stroke has been indicated in previous clinical research [6,7,8,9,10,11,12,13,14,15,16,17]. Some explanations might be given for the impact of hsCRP on recurrence being modulated by stroke severity. Besides vascular inflammation, brain damage and stress were also mainly composed of acute phase inflammation response. It was indicated that the magnitude of acute inflammation response to brain ischemia was related to the volume of infarcted brain. A correlation between CRP and infarct size and severity of stroke per se has been described before [22,23]. Therefore, for the patients with non-minor stroke, it is conceivable that an elevated hsCRP level after stroke could mainly be owing to inflammation related to the pathophysiology of ischemic stroke with a relatively large ischemic area, towering over that triggered by vascular inflammation, the main factor leading to recurrence [44]. Whereas, for minor stroke or TIA with mild ischemia, acute individual inflammation response could be weak, and the increased hsCRP levels may primarily reflect activation of vascular inflammation, which were closely associated with recurrence. This hypothesis was supported by our findings that the association between hsCRP levels and recurrent stroke in the patients with minor stroke or TIA was more apparent in larger-artery atherosclerosis subtype, in line with a prior study which indicated that hsCRP predicted subsequent ischemic events in the patients with intracranial large-artery occlusive disease [7]. Meanwhile, the association between hsCRP and brain ischemic damage maintained regardless of stroke severity, which directly influenced survival. This might be one reason for the persisting significant relationship between hsCRP and death in our study. Though these hypotheses needed to be verified in further studies, our results at least suggested the different mechanisms in hsCRP leading to recurrence and functional outcome.

Thanks to a series of measures, the recurrence rate after minor stroke or TIA declined during the last decade. In the CHANCE trial, the rate dropped from 11.7% to 8.2% by the usage of dual anti-platelet therapy, which was similar to the rate in our study. Combined with the observation that around 98% and 99% patients received anti-platelet agent and statin during follow-up, our data suggested a good adherence to the guideline of secondary prevention in China [45]. However, the residual recurrent risk remained and warranted additional treatment. The effect of anti-inflammation treatment on recurrent cardiovascular disease has been proven recently in large clinical trials [46,47] and is being tested in the patients with non-severe stroke [21]. Our findings help in accurately implying hsCRP to stratify the risk of recurrent stroke, by providing data for potential secondary prevention targets for minor stroke or TIA, as well as in providing evidence in choosing patients for anti-inflammatory therapy.

Though our study derived from a prospective, multicenter, and large sample-sized cohort and centrally-measured blood samples, it also had several limitations. First, the measurement of hsCRP level was only performed at baseline, disenabling us to investigate the effect of hsCRP change on prognosis. Second, though the adherence to secondary prevention was good, information about how the traditional risk factors of blood pressure and blood glucose and lipids levels were controlled during follow-up was unavailable. Third, NIHSS scores often do not reflect the true size of brain lesions. A measurement of brain lesion volume would be more informative. Fourthly, only the Chinese population was enrolled in our study, thus, our findings may not be generalizable to other races and ethnicities.

## 5. Conclusions

Elevated hsCRP levels were related to an increased risk of recurrent stroke in the patients with TIA or minor stroke. However, such a relationship was not found in the patients with non-minor stroke.

## Figures and Tables

**Table 1:** Characteristics according to hsCRP levels among the patients with varying definition of minor stroke or TIA.

	NIHSS ≤ 3 or TIA (*n* = 5790)					NIHSS ≤ 5 or TIA (*n* = 7808)				
	hsCRP Q1	hsCRP Q2	hsCRP Q3	hsCRP Q4	*p* Value	hsCRP Q1	hsCRP Q2	hsCRP Q3	hsCRP Q4	*p* Value
Age (year), median (IQR)	61 (53–68)	61 (54–68)	62 (53–70)	64 (57–73)	<0.0001	61 (53–68)	61 (54–68)	62 (54–70)	65 (57–73)	<0.0001
Male, No. (%)	1198 (72.9)	1133 (69.8)	936 (65.7)	764 (69.5)	0.0004	1574 (72.8)	1493 (69.8)	1288 (66.0)	1062 (68.3)	<0.0001
BMI, median (IQR)	24.2 (22.3–26.0)	24.7 (22.9–26.8)	25.0 (22.9–27.3)	24.7 (22.9–27.0)	<0.0001	24.2 (22.3–26.0)	24.7 (22.9–26.7)	24.9 (22.9–27.2)	24.7 (22.9–26.8)	<0.0001
Smoking, No. (%)	746 (45.4)	750 (46.2)	624 (43.8)	480 (43.7)	0.45	1001 (46.3)	971 (45.4)	869 (44.5)	673 (43.3)	0.28
Medical history, No. (%)										
Ischemic stroke	296 (18.0)	302 (18.6)	256 (18.0)	250 (22.8)	0.007	414 (19.2)	410 (19.2)	380 (19.5)	359 (23.1)	0.01
Diabetes	363 (22.1)	332 (20.5)	370 (26.0)	269 (24.5)	0.002	482 (22.3)	459 (21.5)	518 (26.5)	399 (25.6)	0.0002
Hypertension	967 (58.8)	999 (61.6)	917 (64.4)	724 (65.9)	0.0006	1292 (59.8)	1334 (62.4)	1258 (64.4)	1015 (65.2)	0.002
Hypercholesterolemia	129 (7.9)	136 (8.4)	154 (10.8)	102 (9.3)	0.03	174 (8.1)	175 (8.2)	209 (10.7)	132 (8.5)	0.01
Coronary heart disease	123 (7.5)	128 (7.9)	170 (11.9)	144 (13.1)	<0.0001	176 (8.1)	185 (8.7)	234 (12.0)	198 (12.7)	<0.0001
Atrial fibrillation	57 (3.5)	84 (5.2)	76 (5.3)	100 (9.1)	<0.0001	73 (3.4)	113 (5.3)	103 (5.3)	144 (9.3)	<0.0001
Index event					0.005					0.0004
Ischemic stroke	1401 (85.2)	1410 (86.9)	1268 (89.0)	976 (88.8)		1918 (88.8)	1925 (90.0)	1797 (92.0)	1433 (92.1)	
TIA	243 (14.8)	213 (13.1)	156 (11.0)	123 (11.2)		243 (11.2)	213 (10.0)	156 (8.0)	123 (7.9)	
Baseline NIHSS, median (IQR)	1 (0–2)	1 (0–2)	2 (0–2)	2 (1–2)	0.047	2 (1–3)	2 (1–3)	2 (1–4)	2 (1–4)	<0.0001
Leukocyte count (*10^9^/L), Median (IQR)	6.3 (5.3–7.5)	6.7 (5.6–8.0)	7.0 (5.9–8.7)	7.4 (6.0–9.1)	<0.0001	6.3 (5.3–7.5)	6.7 (5.6–8.0)	7.0 (5.8–8.6)	7.5 (6.1–9.1)	<0.0001
Intravenous rt-PA therapy, No. (%)	82 (5.0)	89 (5.5)	85 (6.0)	67 (6.1)	0.55	127 (5.9)	132 (6.2)	140 (7.2)	122 (7.8)	0.06
Endovascular therapy, No. (%)	2 (0.1)	4 (0.3)	4 (0.3)	10 (0.9)	0.004	3 (0.1)	4 (0.2)	6 (0.3)	14 (0.9)	0.0004
Medication within 1-year follow-up period, No. (%)										
Antiplatelet agents	1627 (99.0)	1606 (99.0)	1399 (98.2)	1087 (98.9)	0.22	2142 (99.1)	2119 (99.1)	1925 (98.6)	1534 (98.6)	0.17
Antihypertensive agents	1016 (61.8)	1051 (64.8)	977 (68.6)	739 (67.2)	0.0005	1342 (62.1)	1393 (65.2)	1337 (68.5)	1057 (67.9)	<0.0001
Hypoglycemic agents	415 (25.2)	431 (26.6)	458 (32.2)	329 (29.9)	<0.0001	554 (25.6)	593 (27.7)	636 (32.6)	499 (32.1)	<0.0001
Statin	1617 (98.4)	1606 (99.0)	1394 (97.9)	1074 (97.7)	0.053	2126 (98.4)	2117 (99.0)	1917 (98.2)	1528 (98.2)	0.10

Abbreviation: IQR = interquartile range; NIHSS = National Institutes of Health Stroke Scale; mRS = modified Rankin Scale; hsCRP = high-sensitive C-reactive Protein; rt-PA = recombinant tissue plasminogen activator. The hsCRP values were categorized into four even levels by quartiles: quartile 1 (Q1): <0.82 mg/L; quartile 2 (Q2): 0.82 to 1.78 mg/L; quartile 3 (Q3):1.78 to 4.72 mg/L; quartile 4 (Q4): >4.72 mg/L.

**Table 2:** Association of hsCRP and recurrent stroke according to severity of cerebrovascular disease.

Group	hsCRP Levels *	Events N (%)	Model 1 ^†^		Model 2 ^‡^		Model 3 ^§^	
			HR (95% CI)	*p* Value	HR (95% CI)	*p* Value	HR (95% CI)	*p* Value
All patients *n* = 10,765	Q1	220 (8.3)	Reference	/	Reference	/	Reference	/
	Q2	230 (8.5)	1.03 (0.86–1.24)	0.73	0.98 (0.81–1.18)	0.81	0.98 (0.80–1.20)	0.85
	Q3	269 (10.0)	1.24 (1.04–1.48)	0.02	1.09 (0.91–1.31)	0.35	1.11 (0.91–1.36)	0.29
	Q4	343 (12.7)	1.62 (1.37–1.92)	<0.0001	1.29 (1.07–1.54)	0.007	1.33 (1.09–1.62)	0.005
	<2 mg/L	480 (8.4)	Reference	/	Reference	/	Reference	/
	≥2 mg/L	582 (11.6)	1.42 (1.26–1.61)	<0.0001	1.22 (1.07–1.39)	0.002	1.25 (1.09–1.44)	0.002
	Continuous model		1.15 (1.10–1.20)	<0.0001	1.08 (1.03–1.13)	0.001	1.10 (1.05–1.16)	<0.0001
NIHSS ≤ 3 or TIA *n* = 5790	Q1	122 (7.4)	Reference	/	Reference	/	Reference	/
	Q2	119 (7.3)	0.99 (0.77–1.28)	0.96	0.98 (0.75–1.27)	0.85	1.01 (0.76–1.34)	0.93
	Q3	127 (8.9)	1.23 (0.96–1.58)	0.10	1.13 (0.87–1.47)	0.35	1.18 (0.89–1.56)	0.25
	Q4	117 (10.7)	1.49 (1.15–1.92)	0.002	1.33 (1.02–1.74)	0.04	1.48 (1.11–1.96)	0.007
	<2 mg/L	260 (7.5)	Reference	/	Reference	/	Reference	/
	≥2 mg/L	225 (9.7)	1.31 (1.10–1.57)	0.003	1.21 (1.003–1.46)	0.046	1.29 (1.05–1.57)	0.01
	Continuous model		1.12 (1.05–1.20)	0.001	1.08 (1.004–1.16)	0.04	1.13 (1.05–1.23)	0.001
NIHSS > 3 *n* = 4975	Q1	98 (9.6)	Reference	/	Reference	/	Reference	/
	Q2	111 (10.2)	1.07 (0.81–1.40)	0.64	0.98 (0.75–1.30)	0.91	0.95 (0.71–1.29)	0.75
	Q3	142 (11.2)	1.19 (0.92–1.54)	0.19	1.03 (0.79–1.34)	0.85	1.03 (0.77–1.37)	0.84
	Q4	226 (14.2)	1.56 (1.23–1.98)	0.0002	1.20 (0.93–1.56)	0.17	1.18 (0.90–1.57)	0.24
	<2 mg/L	220 (9.7)	Reference	/	Reference	/	Reference	/
	≥2 mg/L	357 (13.2)	1.41 (1.19–1.67)	<0.0001	1.19 (0.99–1.43)	0.07	1.19 (0.98–1.45)	0.08
	Continuous model		1.13 (1.07–1.19)	<0.0001	1.06 (1.000–1.13)	0.06	1.07 (1.00–1.14)	0.047
NIHSS ≤ 5 or TIA *n* = 7808	Q1	168 (7.8)	Reference	/	Reference	/	Reference	/
	Q2	176 (8.2)	1.07 (0.86–1.32)	0.55	1.02 (0.82–1.27)	0.85	1.06 (0.84–1.34)	0.63
	Q3	186 (9.5)	1.25 (1.02–1.54)	0.03	1.12 (0.90–1.39)	0.30	1.14 (0.90–1.44)	0.29
	Q4	185 (11.9)	1.59 (1.29–1.96)	<0.0001	1.39 (1.11–1.73)	0.004	1.45 (1.14–1.83)	0.002
	<2 mg/L	369 (8.1)	Reference	/	Reference	/	Reference	/
	≥2 mg/L	346 (10.7)	1.35 (1.17–1.57)	<0.0001	1.22 (1.05–1.43)	0.01	1.24 (1.05–1.46)	0.01
	Continuous model		1.14 (1.08–1.20)	<0.0001	1.09 (1.03–1.16)	0.003	1.13 (1.06–1.20)	0.0002
NIHSS > 5 *n* = 2957	Q1	52 (10.3)	Reference	/	Reference	/	Reference	/
	Q2	54 (9.4)	0.91 (0.62–1.33)	0.62	0.82 (0.56–1.21)	0.31	0.73 (0.48–1.12)	0.15
	Q3	83 (11.3)	1.11 (0.78–1.56)	0.57	0.92 (0.65–1.32)	0.67	0.95 (0.65–1.39)	0.78
	Q4	158 (13.9)	1.42 (1.04–1.94)	0.03	1.01 (0.72–1.41)	0.98	1.01 (0.70–1.46)	0.95
	<2 mg/L	111 (9.6)	Reference	/	Reference	/	Reference	/
	≥2 mg/L	236 (13.2)	1.43 (1.14–1.79)	0.002	1.15 (0.91–1.47)	0.25	1.22 (0.94–1.59)	0.14
	Continuous model		1.12 (1.05–1.20)	0.0008	1.04 (0.96–1.12)	0.36	1.05 (0.96–1.14)	0.30

Patients were classified into minor stroke (NIHSS score ≤3 or ≤5) or TIA, and non-minor stroke (NIHSS score >3 or >5). * The hsCRP values were categorized into four even levels by quartiles: quartile 1 (Q1): <0.82 mg/L; quartile 2 (Q2): 0.82 to 1.78 mg/L; quartile 3 (Q3):1.78 to 4.72 mg/L; quartile 4 (Q4): >4.72 mg/L or two levels according to cut-off level of 2 mg/L. In the continuous model, the hazard ratios correspond to per unit increment of logarithm of the hsCRP value (mg/L). ^†^ Model 1: Unadjusted. ^‡^ Model 2: Adjusted for age, sex, body mass index, smoking history, index event, medical histories of atrial fibrillation, coronary heart disease, ischemic stroke, diabetes, hypertension and hypercholesterolemia, baseline NIHSS score, leukocyte count and low-density lipoprotein cholesterol levels, usage of antiplatelet, antihypertensive, hypoglycemic, and statin during the 1-year follow-up period and TOAST subtype. ^§^ Model 3: Adjusted for all factors in model 2, onset-to-sample collection time, intravenous recombinant tissue plasminogen activator treatment, and endovascular therapy.

**Table 3:** Association of hsCRP and recurrent stroke in large-artery atherosclerosis subtype among the patients with varying definition of minor stroke or TIA.

Group	hsCRP Levels *	Events, No, (%)	Model 1 ^†^		Model 2 ^‡^	
			HR (95% CI)	*p* Value	HR (95% CI)	*p* Value
NIHSS ≤ 3 or TIA *n* = 1265	Q1	32 (10.7)	Reference	/	Reference	/
	Q2	30 (8.8)	0.82 (0.50–1.35)	0.44	0.90 (0.51–1.58)	0.71
	Q3	30 (9.3)	0.89 (0.54–1.46)	0.63	0.97 (0.55–1.71)	0.92
	Q4	50 (16.7)	1.64 (1.05–2.56)	0.03	1.60 (0.95–2.69)	0.08
	<2 mg/L	69 (10.1)	Reference	/	Reference	/
	≥2 mg/L	73 (12.6)	1.28 (0.92–1.78)	0.14	1.24 (0.86–1.80)	0.25
	Continuous model		1.17 (1.04–1.32)	0.01	1.16 (1.00–1.33)	0.049
NIHSS ≤ 5 or TIA *n* = 1752	Q1	40 (9.9)	Reference	/	Reference	/
	Q2	47 (10.0)	1.02 (0.67–156)	0.92	1.21 (0.75–1.96)	0.44
	Q3	45 (9.9)	1.02 (0.66–1.56)	0.94	1.15 (0.70–1.89)	0.57
	Q4	73 (17.2)	1.84 (1.25–2.70)	0.002	1.84 (1.15–2.93)	0.01
	<2 mg/L	96 (10.3)	Reference	/	Reference	/
	≥2 mg/L	109 (13.3)	1.32 (1.01–1.74)	0.05	1.23 (0.89–1.68)	0.21
	Continuous model		1.20 (1.09–1.33)	0.0003	1.20 (1.07–1.35)	0.003

* The hsCRP values were categorized into four even levels by quartiles: quartile 1 (Q1): <0.82 mg/L; quartile 2 (Q2): 0.82 to 1.78 mg/L; quartile 3 (Q3):1.78 to 4.72 mg/L; quartile 4 (Q4): >4.72 mg/L or two levels according to cut-off level of 2 mg/L. In the continuous model, the hazard ratios correspond to per unit increment of logarithm of the hsCRP value (mg/L). ^†^ Model 1: Unadjusted. ^‡^ Model 2: Adjusted for age, sex, body mass index, smoking history, index event, medical histories of atrial fibrillation, coronary heart disease, ischemic stroke, diabetes, hypertension and hypercholesterolemia, baseline NIHSS score, leukocyte count and low-density lipoprotein cholesterol levels, usage of antiplatelet, antihypertensive, hypoglycemic and statin during the 1-year follow-up period, onset-to-sample collection time, intravenous recombinant tissue plasminogen activator treatment, and endovascular therapy.

## Data Availability

All data are stored in China National Clinical Research Center for Neurological Diseases and are available to researchers upon reasonable request by directly contacting the corresponding author.

## Supplementary Materials

The following supporting information can be downloaded at: https://www.mdpi.com/article/10.3390/jcm12041676/s1, Table S1: Distribution of Baseline Characteristics According to Severity of Cerebrovascular Disease; Table S2: Association of hsCRP and Recurrent Stroke in Different TOAST Subtype beyond Large-artery Atherosclerosis Among the Patients with Varying Definition of Minor Stroke or TIA; Table S3: Association of hsCRP and Death according to NIHSS Score.
