# Supplementary material for: Predictive Role of hsCRP in Recurrent Stroke Differed According to Severity of Cerebrovascular Disease: Analysis from a Prospective Cohort Study
Source: J Clin Med. 2023 Feb 20;12(4):1676. doi: 10.3390/jcm12041676 (PMC9967664; doi:10.3390/jcm12041676)
Supplement: Supplementary file 1 [file jcm-12-01676-s001.zip › jcm-2154862-supplementary.pdf]

Supplement Table S1. Distribution of Baseline Characteristics According to Severity of Cerebrovascular Disease.

	All patients n=10765	NIHSS≤3 or TIA n=5790	NIHSS>3 n=4975	P Value	NIHSS≤5 or TIA n=7808	NIHSS>5 n=2957	P Value
Age (year), median (IQR)	63 (54-70)	62 (54-69)	63 (55-71)	<0.0001	62 (54-70)	64 (55-72)	<0.0001
Male, No. (%)	7367 (68.4)	4031 (69.6)	3336 (67.1)	0.004	5417 (69.4)	1950 (66.0)	0.0006
Body mass index, median (IQR)	24.5 (22.6-26.6)	24.5 (22.8-26.7)	24.3 (22.5-26.4)	<0.0001	24.5 (22.8-26.7)	24.2 (22.3-26.2)	<0.0001
Smoking, No. (%)	4789 (44.5)	2600 (44.9)	2189 (44.0)	0.35	3514 (45.0)	1275 (43.1)	0.08
Medical history, No. (%)							
Ischemic stroke	2279 (21.2)	1104 (19.1)	1175 (23.6)	<0.0001	1563 (20.0)	716 (24.2)	<0.0001
Diabetes	2576 (23.9)	1334 (23.0)	1242 (25.0)	0.02	1858 (23.8)	718 (24.3)	0.60
Hypertension	6781 (63.0)	3607 (62.3)	3174 (63.8)	0.11	4899 (62.7)	1882 (63.7)	0.39
Hypercholesterolemia	894 (8.3)	521 (9.0)	373 (7.5)	0.005	690 (8.8)	204 (6.9)	0.001

Coronary heart disease	1164 (10.8)	565 (9.8)	599 (12.0)	0.0001	793 (10.2)	371 (12.6)	0.0004
Atrial fibrillation	766 (7.1)	317 (5.5)	449 (9.0)	<0.0001	433 (5.6)	333 (11.3)	<0.0001
Baseline NIHSS, median (IQR)	3 (1-6)	1 (0-2)	6 (5-9)	<0.0001	2 (1-4)	8 (7-11)	<0.0001
Leukocyte count (*10^9/L), median (IQR)	6.9 (5.7-8.4)	6.8 (5.6-8.2)	7.1 (5.8-8.6)	<0.0001	6.8 (5.7-8.2)	7.3 (5.9-8.9)	<0.0001
LDL-C levels (mmol/L), median (IQR)	2.3 (1.7-3.0)	2.3 (1.7-3.0)	2.4 (1.7-3.0)	0.01	2.3 (1.7-3.0)	2.4 (1.8-3.1)	<0.0001
hsCRP levels, median (IQR)	1.8 (0.8-4.7)	1.4 (0.8-3.5)	2.4 (1.0-6.4)	<0.0001	1.5 (0.8-3.7)	3.0 (1.1-8.4)	<0.0001
Intravenous rt-PA therapy, No. (%)	987 (9.2)	323 (5.6)	664 (13.4)	<0.0001	521 (6.7)	466 (15.7)	<0.0001
Endovascular therapy, No. (%)	77 (0.7)	20 (0.4)	57 (1.2)	<0.0001	27 (0.4)	50 (1.7)	<0.0001
Medication within 1-year follow-up period, No. (%)							
Antiplatelet agents	10595 (98.4)	5719 (98.8)	4876 (98.0)	0.002	7720 (98.9)	2875 (97.2)	<0.0001
Antihypertensive agents	7019 (65.2)	3783 (65.3)	3236 (65.1)	0.75	5129 (65.7)	1890 (63.9)	0.08
Hypoglycemic agents	3171 (29.5)	1633 (28.2)	1538 (30.9)	0.002	2282 (29.2)	889 (30.1)	0.39

Statin	10601 (98.5)	5691 (98.3)	4910 (98.7)	0.09	7688 (98.5)	2913 (98.5)	0.85

Patients were classified into minor stroke (NIHSS score ≤3 or ≤5) or TIA, and non-minor stroke (NIHSS score >3 or >5).

Abbreviation: IQR = interquartile range; NIHSS=National Institutes of Health Stroke Scale; mRS=modified Rankin Scale; LDL-C=low-density lipoprotein cholesterol; hsCRP=high-sensitive C-reactive Protein; rt-PA=recombinant tissue plasminogen activator.

Supplement Table S2. Association of hsCRP and Recurrent Stroke in Different TOAST Subtype beyond Large-artery Atherosclerosis Among the Patients with Varying Definition of Minor Stroke or TIA.

Subtype	Group	hsCRP levels*	Events, No, (%)	Model 1 †		Model 2 ‡	
				HR (95% CI)	P Value	HR (95% CI)	P Value
Cardioembolism n=693	NIHSS≤3 or TIA n=319	Q1	6 (8.5)	Reference	/	Reference	/
		Q2	7 (8.0)	0.94 (0.32-2.81)	0.92	0.95 (0.31-2.90)	0.93
		Q3	14 (18.2)	2.27 (0.87-5.90)	0.09	2.04 (0.76-5.50)	0.16
		Q4	6 (7.2)	0.86 (0.28-2.67)	0.79	0.67 (0.20-2.27)	0.52
		<2 mg/L	14 (8.2)	Reference	/	Reference	/
		≥2 mg/L	19 (12.8)	1.57 (0.79-3.14)	0.20	1.41 (0.67-2.96)	0.36
		Continuous model		1.02 (0.79-1.31)	0.88	0.95 (0.72-1.25)	0.72
	NIHSS≤5 or TIA	Q1	7 (7.7)	Reference	/	Reference	/

	n=435	Q2	13 (11.0)	1.47 (0.59-3.69)	0.41	1.62 (0.63-4.11)	0.31
		Q3	15 (14.0)	1.89 (0.77-4.64)	0.16	1.79 (0.71-4.50)	0.22
		Q4	16 (13.5)	1.82 (0.75-4.43)	0.19	1.86 (0.72-4.83)	0.20
		<2 mg/L	22 (9.8)	Reference	/	Reference	/
		≥2 mg/L	29 (13.8)	1.43 (0.82-2.49)	0.20	1.32 (0.73-2.37)	0.36
		Continuous model		1.11 (0.92-1.34)	0.29	1.10 (0.89-1.37)	0.37
Small-vessel occlusion n=2242	NIHSS≤3 or TIA n=1346	Q1	30 (6.7)	Reference	/	Reference	/
		Q2	23 (5.8)	0.86 (0.50-1.49)	0.60	0.79 (0.44-1.43)	0.44
		Q3	23 (7.4)	1.11 (0.64-1.90)	0.72	1.02 (0.55-1.87)	0.96
		Q4	8 (4.3)	0.65 (0.30-1.41)	0.27	0.64 (0.28-1.46)	0.29
		<2 mg/L	55 (6.1)	Reference	/	Reference	/
		≥2 mg/L	29 (6.6)	1.10 (0.70-1.72)	0.69	1.10 (0.67-1.82)	0.71

		Continuous model		0.90 (0.74-1.10)	0.29	0.89 (0.71-1.11)	0.31
	NIHSS≤5+TIA n=1818	Q1	45 (7.4)	Reference	/	Reference	/
		Q2	31 (6.0)	0.80 (0.51-1.27)	0.34	0.73 (0.45-1.18)	0.20
		Q3	29 (6.8)	0.92 (0.58-1.46)	0.72	0.79 (0.47-1.32)	0.37
		Q4	20 (7.6)	1.05 (0.62-1.78)	0.85	0.93 (0.53-1.65)	0.81
		<2 mg/L	78 (6.5)	Reference	/	Reference	/
		≥2 mg/L	47 (7.7)	1.21 (0.84-1.73)	0.31	1.12 (0.75-1.67)	0.59
		Continuous model		0.99 (0.85-1.15)	0.86	0.94 (0.79-1.12)	0.47
Stroke of other determined etiology n=120	NIHSS≤3 or TIA n=68	Q1	0 (0)	Reference	/	Reference	/
		Q2	2 (10.5)	/	/	/	/
		Q3	3 (14.3)	/	/	/	/
		Q4	2 (22.2)	/	/	/	/

		<2 mg/L	2 (5.0)	Reference	/	Reference	/
		≥2 mg/L	5 (17.9)	3.86 (0.75-19.9)	0.11	4.21 (0.46-38.4)	0.20
		Continuous model		1.49 (1.01-2.20)	0.04	3.81 (1.06-13.7)	0.04
	NIHSS≤5 or TIA n=88	Q1	1 (4.2)	Reference	/	Reference	/
		Q2	2 (8.3)	1.97 (0.18-21.7)	0.58	1.43 (0.09-22.7)	0.80
		Q3	3 (11.5)	2.80 (0.29-26.9)	0.37	1.41 (0.08-.26.7)	0.82
		Q4	2 (14.3)	3.52 (0.32-38.8)	0.30	1.94 (0.07-57.2)	0.70
		<2 mg/L	3 (6.0)	Reference	/	Reference	/
		≥2 mg/L	5 (13.2)	2.26 (0.54-9.46)	0.26	1.60 (0.30-8.64)	0.58
		Continuous model		1.36 (0.92-2.03)	0.13	1.33 (0.71-2.49)	0.37
Stroke of undetermined	NIHSS≤3 or TIA n=2792	Q1	54 (6.7)	Reference	/	Reference	/
		Q2	57 (7.4)	1.11 (0.76-1.61)	0.59	1.17 (0.79-1.71)	0.44

etiology n=5002		Q3	57 (8.3)	1.27 (0.87-1.84)	0.22	1.18 (0.80-1.75)	0.40
		Q4	51 (9.8)	1.52 (1.03-2.22)	0.03	1.45 (0.97-2.16)	0.07
		<2 mg/L	120 (7.2)	Reference	/	Reference	/
		≥2 mg/L	99 (8.7)	1.24 (0.95-1.61)	0.12	1.16 (0.88-1.53)	0.30
		Continuous model		1.12 (1.01-1.24)	0.03	1.09 (0.98-1.22)	0.11
	NIHSS≤5 or TIA n=3715	Q1	75 (7.3)	Reference	/	Reference	/
		Q2	83 (8.3)	1.15 (0.84-1.57)	0.38	1.15 (0.83-1.58)	0.41
		Q3	94 (10.0)	1.42 (1.05-1.93)	0.02	1.28 (0.93-1.76)	0.13
		Q4	74 (10.1)	1.44 (1.04-1.98)	0.03	1.30 (0.93-1.83)	0.12
		<2 mg/L	170 (7.9)	Reference	/	Reference	/
		≥2 mg/L	156 (10.0)	1.30 (1.04-1.61)	0.02	1.19 (0.94-1.49)	0.14
		Continuous model		1.11 (1.02-1.20)	0.02	1.07 (0.98-1.17)	0.13

*hsCRP values were categorized into 4 even levels by quartiles: quartile 1 (Q1): <0.82mg/L; quartile 2 (Q2): 0.82 to 1.78 mg/L; quartile 3 (Q3):1.78

to 4.72 mg/L; quartile 4 (Q4): >4.72 mg/L or 2 levels according to cut-off level of 2mg/L. In the continuous model, the hazard ratios correspond to per unit increment of logarithm of hsCRP value (mg/L).

† Model 1: Unadjusted;

‡ Model 2: Adjusted for age, sex, body mass index, smoking, index event, medical histories of atrial fibrillation, coronary heart disease, ischemic stroke, diabetes, hypertension and hypercholesterolemia, baseline NIHSS score, leukocyte count and low-density lipoprotein cholesterol levels and usage of antiplatelet, antihypertensive, hypoglycemic and statin during 1-year follow-up period.

Supplement Table S3. Association of hsCRP and Death according to NIHSS Score.

Group	hsCRP levels	Events, No, (%)	Model 1 †		Model 2 ‡	
			HR (95% CI)	P Value	HR (95% CI)	P Value
All Patients	Q1	46 (1.7)	Reference	/	Reference	/
	Q2	46 (1.7)	0.99 (0.66-1.49)	0.96	0.85 (0.54-1.33)	0.47
	Q3	64 (2.4)	1.40 (0.96-2.04)	0.08	1.06 (0.71-1.60)	0.77
	Q4	205 (7.6)	4.62 (3.36-6.36)	<0.0001	2.07 (1.44-2.98)	<0.0001
	<2 mg/L	102 (1.8)	Reference	/	Reference	/
	≥2 mg/L	259 (5.1)	2.96 (2.35-3.72)	<0.0001	1.64 (1.26-2.13)	0.0002
	Continuous model		1.65 (1.56-1.76)	<0.0001	1.31 (1.22-1.41)	<0.0001
NIHSS≤5 or TIA	Q1	32 (1.5)	Reference	/	Reference	/
	Q2	25 (1.2)	0.79 (0.47-1.34)	0.39	0.67 (0.37-1.21)	0.18

n=7808	Q3	35 (1.8)	1.23 (0.76-1.98)	0.41	1.10 (0.66-1.84)	0.72
	Q4	71 (4.6)	3.18 (2.09-4.83)	<0.0001	1.88 (1.16-3.04)	0.01
	<2 mg/L	63 (1.4)	Reference	/	Reference	/
	≥2 mg/L	100 (3.1)	2.27 (1.66-3.11)	<0.0001	1.66 (1.16-2.37)	0.006
	Continuous model		1.52 (1.38-1.67)	<0.0001	1.31 (1.17-1.47)	<0.0001
	Q1	14 (2.8)	Reference	/	Reference	/
	Q2	21 (3.7)	1.34 (0.68-2.64)	0.40	1.13 (0.54-2.36)	0.75
	Q3	29 (4.0)	1.44 (0.76-2.72)	0.26	0.97 (0.48-1.96)	0.94
NIHSS>5 n=2957	Q4	134 (11.8)	4.53 (2.61-7.85)	<0.0001	2.10 (1.12-3.92)	0.02
	<2 mg/L	39 (3.4)	Reference	/	Reference	/
	≥2 mg/L	159 (8.9)	2.73 (1.92-3.88)	<0.0001	1.43 (0.96-2.13)	0.08
	Continuous model		1.58 (1.45-1.72)	<0.0001	1.29 (1.16-1.43)	<0.0001

* All markers were categorized into 4 even groups by quartiles: quartile 1 (Q1): <0.82mg/L; quartile 2 (Q2): 0.82 to 1.78 mg/L; quartile 3 (Q3):1.78 to 4.72 mg/L; quartile 4 (Q4): >4.72 mg/L or 2 levels according to cut-off level of 2mg/L. In the continuous model, the hazard ratios correspond to per unit increment of logarithm of hsCRP value (mg/L).

† Model 1: unadjusted.

‡ Model 2: adjusted for age, sex, body mass index, smoking, index event, medical histories of atrial fibrillation, coronary heart disease, ischemic stroke, diabetes, hypertension and hypercholesterolemia, baseline NIHSS score, mRS score before the onset of index events and baseline leukocyte count, intravenous recombinant tissue plasminogen activator treatment, endovascular therapy, usage of antiplatelet, antihypertensive, hypoglycemic and statin during 1-year follow-up period, stroke recurrence within 1 year, TOAST subtype and symptom onset-sampling time.
